# The Importance of Gut Symbionts in the Development of the Brown Marmorated Stink Bug, *Halyomorpha halys* (Stål)

**DOI:** 10.1371/journal.pone.0090312

**Published:** 2014-03-05

**Authors:** Christopher M. Taylor, Peter L. Coffey, Bridget D. DeLay, Galen P. Dively

**Affiliations:** University of Maryland, Department of Entomology, College Park, Maryland, United States of America; Queensland Institute of Medical Research, Australia

## Abstract

The invasive brown marmorated stink bug, *Halyomorpha halys* (Stål), has become a severe agricultural pest and nuisance problem since its introduction in the U.S. Research is being conducted to understand its biology and to find management solutions. Its symbiotic relationship with gut symbionts is one aspect of its biology that is not understood. In the family Pentatomidae, the reliance on gut symbionts for successful development seems to vary depending on the species of stink bug. This research assessed the role of gut symbionts in the development, survivorship, and fecundity of *H. halys*. We compared various fitness parameters of nymphs and adults reared from surface sterilized and untreated egg masses during two consecutive generations under laboratory conditions. Results provided direct evidence that *H. halys* is negatively impacted by the prevention of vertical transmission of its gut symbionts and that this impact is significant in the first generation and manifests dramatically in the subsequent generation. Developmental time and survivorship of treated cohorts in the first generation were significantly affected during third instar development through to the adult stage. Adults from the sterilized treatment group exhibited longer pre-oviposition periods, produced fewer egg masses, had significantly smaller clutch sizes, and the hatch rate and survivorship of those eggs were significantly reduced. Observations following hatch of surface sterilized eggs also revealed significant effects on wandering behavior of the first instars. The second generation progeny from adults of the sterilized cohorts showed significantly lower survival to adulthood, averaging only 0.3% compared to 20.8% for the control cohorts. Taken together, results demonstrate that *H. halys* is heavily impacted by deprival of its gut symbionts. Given the economic status of this invasive pest, further investigations may lead to management tactics that disrupt this close symbiotic relationship in the biology of *H. halys*.

## Introduction

The brown marmorated stink bug, *Halyomorpha halys* (Stål), is a highly polyphagous pest species [1] indigenous to northeastern Asia where it damages various trees, vegetables, and leguminous crops [2]. It was first identified as an invasive pest in the U.S. in 2001 but previous reports of its presence in eastern Pennsylvania date back to 1996 [2]. Since its introduction, *H. halys* has rapidly expanded its range and has now been detected in 40 states [3]. Populations have steadily increased in the northeast U.S., where it has become a severe agricultural pest in six states [4], [5] and a nuisance problem in 13 states due to its en masse overwintering habits in structures [1], [6]. As an invasive species, there is limited information on H. halys and its impacts on U.S. agriculture and the environment in North America. With funding from USDA and commodity organizations, several working groups of more than 60 researchers are conducting a diverse range of studies to understand the biology and ecology of this pest and to find management solutions.

Like many insects in the order Hemiptera that share symbiotic relationships with microorganisms, *H. halys* is presumed to share a close symbiotic relationship with gut flora. Newly hatched nymphs aggregate and stay closely associated with the egg mass for several days, probing the egg chorion for the symbionts which are thought to colonize the sterile caeca of the midgut. The importance of the gut symbionts to the development and survival of *H. halys* is not known. Symbiotic relationships in the Hemiptera are well documented in the Auchenorrhyncha and Sternorrhyncha and are typically found within the diet specific Heteropteran infraorders Cimicomorpha (namely the sanguinivorous bed bugs [Cimicidae] and assassin bugs [Reduviidae: *Triatoma*]) and Pentatomomorpha, which includes many agriculturally important families such as the stink bugs (Pentatomidae), shield bugs (Scutelleridae), and plataspids (Plataspidae) [7]. The gut symbionts are essential for maintaining the fitness of many hemipterans that have been studied because they provide the amino acids and vitamins necessary for development that are lacking in phloem sap [8], [9], [10]. The bacteria are housed within the gastric caeca in the Pentatomomorpha families [7], [11], [12], although the method by which vertical transmission occurs varies. For some, such as the family Plataspidae, bacteria-containing capsules are deposited with the egg mass and nymphs probe these capsules to become inoculated [7], [13], [14]. Some nymphs will probe the symbiont rich fecal material of the adults to become inoculated, as seen in the family Cydnidae [15]. For the Pentatomidae and related families, nymphs probe the egg mass which is coated with the symbiont by the mother [7], [16], [17], [18], [19]. This ‘post hatch’ inoculation behavior is believed to be due to the extracellular nature of the symbiont housing within the gut [20].

Sterilization has been used in studies to prevent the nymphs from acquiring the deposited gut symbionts in order to study the effects of symbiont deprivation [13], [17], [21]. Some studies have demonstrated that experimental removal of the symbiont can lead to negative impacts on the hemipteran host, such as a decrease in fitness, slowed development and/or higher nymph mortality (e.g. Plataspidae: [13]; Pentatomidae: [16], [21], [22]; Parastrachiidae: [23]; Cydnidae: [15]; Acanthosomatidae: [18]). In a very specialized case, the subsocial *Parastrachia japonensis* females care for the eggs and secrete symbiont rich mucus less than an hour before synchronous nymphal hatch. When females are removed, vertical symbiont transmission is prevented and eggs hatch asynchronously [24]. However the patterns of negative impacts on the host after removal of the symbiont are not universally true and it appears that there are varying degrees of reliance on the symbiont by its host, at least within the Pentatomidae. Surface sterilization experiments showed no difference in developmental time over two generations in the southern green stink bug *Nezara viridula* [17]. The harlequin bug *Murgantia histrionica* saw a longer generation time but a slight increase in survivorship when its symbionts were removed [21]. Environmental factors may also play a role, as high temperature was shown to negatively affect the symbionts and was coupled with a decrease in fitness in *M. histrionica* and the green stink bug *Chinavia hilaris* (formerly *Acrosternum hilare*) [25]. For *C. hilaris*, the symbiont is required for adequate development [21], which suggests that temperature effects on the symbionts can negatively impact the host in these cases.

It is apparent that there is no distinct trend available to gauge the level of dependency on gut symbionts in the Pentatomidae. Although most stink bug symbionts were found to belong to the clade to which the bacterial genera *Erwinia* and *Pantoea* belong, the relationships between them are complicated and symbiont monophyly doesn't occur above the pentatomid genus level [26]. This complexity is seen in other Heteropteran groups as well, such as the families Cydnidae [27] and Scutelleridae [28]. Therefore, these symbiotic relationships must be studied on a species to species basis. The purpose of this research was to assess the role of gut symbionts on the development, survivorship and fecundity of *H. halys*. We compared various fitness parameters of nymphs and adults reared from surface sterilized and untreated egg masses under laboratory conditions, and determined the importance of the gut symbionts. An understanding of the role and complexities of symbiotic relationships in the Heteroptera could lead to possible exploitation as a strategy for managing pestiferous species like *H. halys* [29].

## Materials and Methods

### Ethics Statement

The wild caught *Halyomorpha halys* used to start the lab colony before the experiment were collected in soybean fields at the University of Maryland Beltsville Research Farm where we have permission to conduct research and collect samples. No specific permission was required to collect *H. halys*, and no endangered or protected species were involved.

### Insect culture

A laboratory colony of *H. halys* was established in January 2012 with adults collected from soybean fields at our University of Maryland Beltsville Research Farm during the previous fall and held for several months at 12±0.5°C to break diapause. The insects were reared on potted plants of *Phaseolus vulgaris*, excised bean pods, and raw sunflower seeds in mesh cages (60×30×35 cm). The colony was maintained for three generations in walk-in environmental chambers at 25°C, RH of 65±5%, and a 16 h L: 8 h D photoperiod. Egg masses laid within 24 hours were collected from bean plants from several cages of adults exhibiting peak oviposition. A 15 mm leaf disc containing each egg mass was cut from the bean leaves using a cork borer.

### Egg mass treatments

Thirty-six egg masses, each consisting of 28 eggs representing the median clutch size [30] were randomly divided into two treatment groups. One group was surface sterilized in 10% Clorox solution to remove the symbionts present. To determine the maximum soaking time for *H. halys* egg masses, we conducted a preliminary experiment involving groups of egg masses soaked for either 2, 4, 6, or 8 minutes in 10% Clorox solution. Results showed that 6 minutes in the bleach solution was the longest soaking time allowed before *H. halys* eggs began to dissociate from their egg mass, which can negatively impact hatch rate [31]. Using this soaking time and a modified protocol by Prado and Almeida [17], [21], each egg mass was submerged in a 100% ethanol bath for 5 minutes; then submerged in a 10% Clorox bleach solution for 6 minutes; and then rinsed, first in a separate ethanol bath and then in a distilled water bath. The control group of 18 egg masses was not manipulated in any way.

### Nymphal rearing (generation 1)

Each egg mass was reared in a clear (16×14×5 cm) plastic deli dish, with a screened opening (8 cm diameter) in the lid to provide ventilation. Each dish contained an excised organic bean pod and a 15 ml floral water pick to provide a transportable water supply for an inserted leaf terminal of *P. vulgaris*. The deli dishes were held in an environmental chamber set at 25°C, 65–75% humidity, and a 16 h L: 8 h D diurnal cycle. To account for possible environmental gradients within the chamber, six cohorts of each treatment were randomly assigned to each of the three shelves which were treated as a blocking factor. The dishes were lightly misted with distilled water every day, and fresh leaf terminals and bean pods were provisioned every 4–6 days. Data were recorded each day on the number of live stink bugs of each developmental instar. Dead insects were also recorded and removed each day. Time expressed in developmental days was recorded when each egg mass hatched (Day 1) to synchronize development across all cohorts.

### Instar behavioral observations (generation 1)

Because symbiont presence or absence may influence the behavior of newly-hatched nymphs [32], the behavior of the first instars was recorded until molting to the second instar began. The number of nymphs clustered around and physically touching the egg mass compared to the number wandering was recorded for each cohort during days 1–5. Nymphs were not manipulated or prodded to prevent behavior modification during this time.

### Adult rearing (generation 1)

Adult stink bugs produced in the replicate cohorts of each treatment were collected and either stored for PCR analysis or transferred to larger rearing cages to monitor survival and oviposition. It was not possible to maintain the replication of individual cohorts, so adults were pooled from the six cohorts of each treatment that were reared on each shelf of the rearing chamber. Because there was a significant blocking effect, nymphs in cohorts reared on the lowest shelf produced insignificant numbers of adults for further testing, although the control cohorts still performed better than the sterilized cohorts. Thus, only four rearing cages were established by pooling adults collected from the six cohorts of each treatment from the upper two shelves. This was necessary to avoid overcrowding in the rearing cages, as well as to track any possible blocking effect that might carry over to the second generation.

Adults were reared under the same environmental conditions in the same mesh cages as described above for the laboratory colony. Records were kept each day on the number of new adults added and the total number of adults per cage. Dead adults were also recorded and removed daily. Similar to colony rearing, cages were provisioned with six potted bean plants, bean pods, and shelled sunflower seeds to provide an additional protein source. Plants and supplemental foods were replaced every 4–6 days as needed.

### Egg mass selection and nymphal rearing (generation 2)

Plants and mesh surfaces of the adult cages were checked daily for egg masses. Because developmental time to adulthood varied among replicate cohorts, new adults were added to the rearing cages over the course of several weeks. Subsequently, egg masses were produced continuously in the rearing cages, once *H. halys* females passed the 2 week pre-oviposition period [30]. Adults from the control group produced considerably more eggs over time than adults from the sterilized group. To provide a representative selection of egg masses for generation 2 rearing, only one egg mass was chosen every other day from each control cage until 12 masses were collected. Ultimately, a total of 24 replicate egg masses from the control group were selected for generation 2 rearing. Both cages of adults reared from sterilized eggs produced only 15 egg masses total and all were chosen for generation 2 rearing. Each egg mass on a 15 mm leaf disc was placed in a deli dish containing a bean pod and leaf terminal of *P. vulgaris*, and maintained following the same protocol used for generation 1 rearing. No manipulation of the egg masses was performed for either treatment. To account for possible environmental gradients in rearing, six deli dishes of each treatment group were placed on each of two shelves in two separate environmental chambers based on the shelf from which the adults that laid them came. Similar to the data recorded in generation 1, daily records for each cohort were kept on the number of live and dead nymphs of each developmental instar. To track and compare development of cohorts since they were set up over several weeks in each treatment group, day 1 in developmental time commenced when each egg mass hatched like in generation 1. All other egg masses from the control cages that were not used for generation 2 rearing were also collected and hatched in deli dishes to provide additional data on the time to hatch, clutch size, and level of hatchability.

### PCR Analysis

Prior studies isolated two symbionts from gut dissections of field caught *H. halys* adults and primers were developed for their detection (unpublished data). One symbiont was a species of *Wolbachia*, while the second was *Pantoea agglomerans*. PCR detection of the *Wolbachia* symbiont was unsuccessful on multiple trials, but was successful for *P. agglomerans*.

To verify the presence of *Wolbachia* and *P. agglomerans* in adults and egg masses from the colony, samples of both life stages were preserved in 100% ethanol-filled centrifuge tubes and stored for later DNA extraction. To substantiate whether egg masses were successfully sterilized and the sterilization effect carried over to the adult stage, subsamples of male and female adults from treatment groups of both generations, as well as remains of every hatched egg mass from the treatment groups were also stored for DNA extraction. All samples were extracted for DNA using a Quiagen DNEasy Blood and Tissue Kit. The entire egg mass or remains was used for extraction, while only the abdomen and gut contents of adult stink bugs were removed and well macerated in the collection tube prior to DNA extraction to allow for optimal lysing. Each individual adult was analyzed separately and gel band presence or absence was determined for each. We also used a NanoDrop Spectrophotometer to analyze DNA concentrations of a subset of adult and egg mass husk extractions to determine whether adequate amounts of DNA were extracted for PCR. This was mainly done for the egg mass husks, which may have been devoid of microbes by the time the nymphs had moved off of them and they were stored for analysis.

### Data analysis

PCR data analysis between treatments was limited due to sample size, but a One-Tailed Fishers Exact Test was used to analyze differences between generation 1 adults by treatment. The effect of sterilization on nymph and adult development of *H. halys* in days from egg hatch was tested for each generation using a one-way analysis of variance (Proc Mixed, SAS). For survivorship, a two way ANOVA was used to analyze the treatment, generation and interaction effects on each stage, after an arcsine square-root transformation of the percentage data. In both analyses, each stage was analyzed separately. Several variables of egg mass production (egg masses per female, clutch size, days to hatch, and percent hatchability) were analyzed separately using a one-way ANOVA. The behavior movement of first instar from the egg mass was tested for both treatment and treatment by time effects after adjustments were made for auto-correlation between days. All count data were tested for lack of normality and either log- or square root-transformed prior to statistical analyses. In certain analyses, a random blocking factor was included in the model to remove experimental variance due to the position of the deli dishes within the rearing chambers. For mean comparisons, Tukey-Kramer's test was used to calculate adjusted *P* values for differences.

## Results

### Spectrophotometer and PCR Analysis

PCR detection of the *Wolbachia* symbiont was unsuccessful on multiple trials. From this point forward, all reference to the symbiont refers to the second species, *P. agglomerans* unless otherwise stated. Colony adults showed a DNA concentration between 190–240 ng/µl and strong bands were detected on the running gel for *P. agglomerans*. For untreated egg masses collected directly from the colony, one sample showed a 90 ng/µl concentration and a weak gel band, while the other showed a −1.1 ng/µl concentration with no band for *P. agglomerans*. These results suggested that individual egg masses might not be good indicators of symbiont presence or absence. To maximize DNA yield for DNA extraction, we pooled chorions of hatched egg masses by block for both treatment groups of generation 1. However, even with pooling and a lower final elution amount to increase DNA concentration, all samples showed between 0 to 8 ng/µl of DNA. We then used PCR to test for the presence of *P. agglomerans* on unhatched control and sterilized egg masses pooled in groups of two to increase DNA yield. Using lower elution amount during the extraction process, control egg masses showed distinctly bright gel bands for the symbiont, which confirmed its presence on egg masses, whereas the sterilized egg masses showed qualitatively weak gel bands, indicating that either some bacterial cells survived the sterilization treatment, or residual bacterial DNA was not completely rinsed off of the egg masses (and trapped between eggs). Based on these findings, PCR was not performed on the generation 1 and 2 egg mass chorions because we concluded that they lacked sufficient DNA for accurate PCR detection.

For the adult extractions, 21 of the 24 (87.5%) control adults of the first generation tested positively, while only 7 of 18 (38.9%) tested positively in the sterilized treatment group. This difference in adult inoculation rates between treatment groups was significant (*P*<0.001, One-Tailed Fisher's Exact Test). For the second generation, since survivorship to adults was very low in the sterilized treatment, we conducted PCR analysis on only one surviving adult but also tested samples of individual 2^nd^ and 5^th^ instar nymphs from the sterilized group. Of these samples, the adult, 2 of the four 5^th^ instars, and 3 of the six 2^nd^ instars tested positive for *P. agglomerans*, although the gel bands were qualitatively weak in all positive samples. Eight randomly selected generation 2 adults from the control treatment were selected to verify carryover from generation 1, and all tested positively for *P. agglomerans*.

### Developmental time

**Figure 1:**
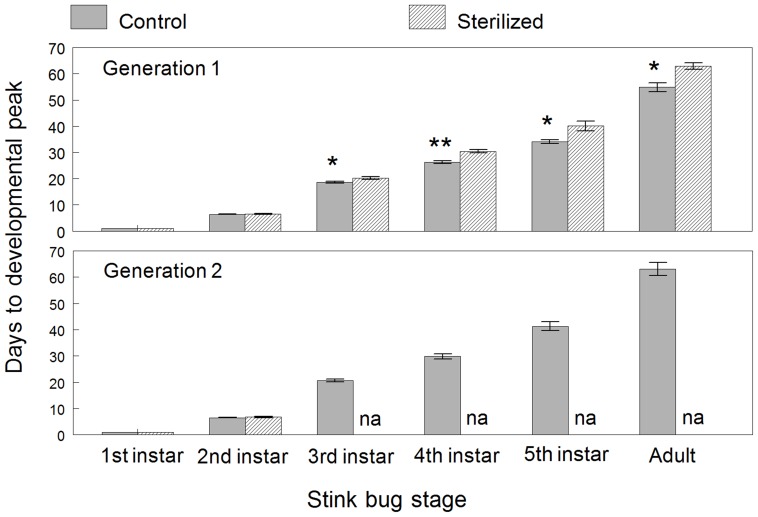
Development time expressed as the number of days from egg hatch required to reach peak density of each stage of *H. halys*. Means (± SEM) are plotted for first generation cohorts reared from sterilized and untreated control eggs and for subsequent second generation cohorts. An asterisk over each pair of means for each developmental stage indicates a significant difference (*P*<0.05); two asterisks indicate a very significant difference (*P*<0.001). ‘NA’ indicates that no data were available.

Since the exact age of egg masses was unknown (all laid within 24 hours) and most neonates hatched in synchrony within a few hours, exact egg development prior to hatch could not be determined. For the subsequent nymphal instars and adults, development time was expressed as the number of days from egg hatch (Day 1) that was required to reach the peak density of individuals of each stage. ANOVA results showed that the surface sterilization treatment resulted in an overall delay in development, except for development to the second instar (Figure 1, Table 1). Nymphs from sterilized egg masses took significantly longer to develop to the third instar than nymphs from untreated control eggs, and differences in development times significantly increased with each subsequent life stage. Time to peak numbers of adults from sterilized eggs averaged 62.9 days from egg hatch, 8.1 days later than the control group.

**Table 1:** Developmental time by life stage for Generation 1.

*Developmental stage*	*Control group* *Mean (days)*	*Sterile group Mean (days)*	*Df*	*F*	*P*
**1^st^ instar**	NA	NA	-	-	-
**2^nd^ instar**	6.4±.12	6.5±.12	1, 32	0.46	0.50
**3^rd^ instar**	18.6±.34	20.2±.54	1, 31	11.69	0.002
**4^th^ instar**	26.2±.55	30.4±.60	1, 29	32.59	<0.001
**5^th^ instar**	34.1±.73	40.1±1.83	1, 29	11.31	0.002
**Adult (males)**	50.6±1.52	60.5±1.86	1, 23	16.45	<0.001
**Adult (females)**	51.5±2.17	59.3±1.51	1, 24	6.73	0.016
**Adult (total)**	54.8±1.65	62.9±1.31	1, 27	11.77	0.002

The same analysis was performed on the of development time of generation 2 nymphs hatching from egg masses produced by adults from the control and sterilized groups in generation 1. These eggs were not manipulated in any way. However, few progeny from the sterilized adults survived after the second instar. Only the data on development of the second instars was available for statistical analysis, which showed no difference between cohorts that developed from egg masses laid by adults from the control and sterilized first generation cohorts (Figure 1, Table 2).

**Table 2:** Developmental time by life stage for Generation 2.

*Developmental stage*	*Control group* *Mean (days)*	*Sterile group Mean (days)*	*Df*	*F*	*P*
**1^st^ instar**	NA	NA	-	-	-
**2^nd^ instar**	6.5±.12	6.8±.25	1, 31	1. 25	0.27

### Survivorship

**Figure 2:**
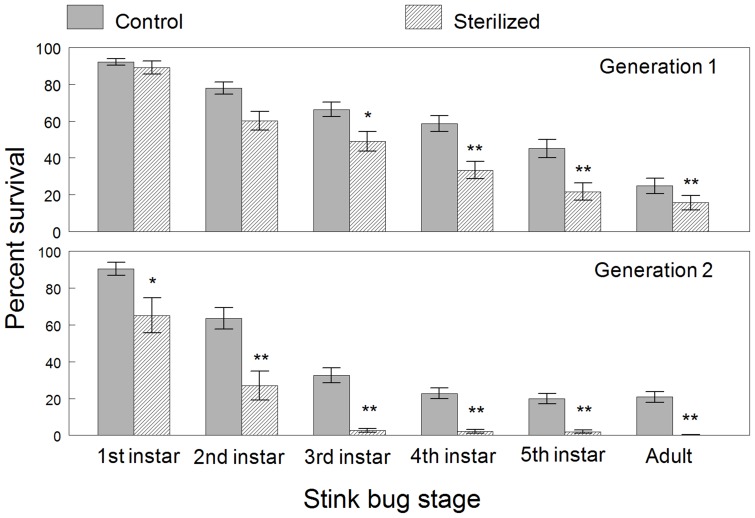
Percent survival from egg hatch to the peak density of each developmental stage of *H. halys* during two successive generations. Means (± SEM) are plotted for first generation cohorts reared from sterilized and untreated control eggs and for subsequent second generation cohorts. An asterisk over each pair of means for each developmental stage indicates a significant difference (*P*<0.05); two asterisks indicate a very significant difference (*P*<0.001).

The percent survival to each nymph instar and adult stage was calculated by dividing the total number of eggs per mass in each cohort by the peak density of individuals that reached each developmental stage. This value was considered a relative estimate of survival since development through the instars was continuous and thus the peak density may be less than the absolute numbers of individuals going through the developmental stage. Nevertheless, the relative estimates given in Table 3 and Figure 2 provided a means to compare survival between treatments and generations.

**Table 3:** Survivorship by life stage for both generations.

	*First generation survivorship*		*Second generation survivorship*					
*Life Stage*	*Mean % control group*	*Mean % sterile group*	*Mean % control group*	*Mean % sterile group*	*Effect*	*Df*	*F*	*P*
**1^st^**	92.3±1.66	89.3±3.53	90.5±3.54	65.2±9.52	T	1, 71	7.92	0.006
**1^st^**					G	1, 71	4.31	0.042
**1^st^**					T x G	1, 71	5.92	0.018
**2^nd^**	78.0±3.32	60.3±5.01	63.5±5.75	27.0±7.97	T	1, 71	22.96	<0.001
**2^nd^**					G	1, 71	18.73	<0.001
**2^nd^**					T x G	1, 71	3.62	0.061
**3^rd^**	66.4±4.01	48.9±5.36	32.6±4.04	2.7±1.09	T	1, 69	39.65	<0.001
**3^rd^**					G	1, 62	77.73	<0.001
**3^rd^**					T x G	1, 69	5.82	0.019
**4^th^**	58.6±4.38	33.4±4.76	22.8±2.87	2.2±1.05	T	1, 69	58.54	<0.001
**4^th^**					G	1, 70	112.5	<0.001
**4^th^**					T x G	1, 69	0.71	0.40
**5^th^**	45.2±4.99	21.6±4.71	19.9±2.83	2.0±0.93	T	1, 69	63.5	<0.001
**5^th^**					G	1, 70	83.49	<0.001
**5^th^**					T x G	1, 69	0.38	0.54
**Adult**	24.8±4.16	15.7±3.98	20.8±2.90	0.3±0.26	T	1, 69	57.96	<0.001
**Adult**					G	1, 70	42.25	<0.001
**Adult**					T x G	1, 69	11.45	0.001

Footnote: Two-way ANOVA with percent survival as the dependent variable, egg mass treatment (T), generation (G), and the interaction (T x G) as fixed factors, and position in the rearing chamber as a random blocking factor.

Survival to the first instar was indicated by the mean percentage of egg hatch which ranged from 89 to 92% in generation 1 and was not significantly different between treatments (*t*_(1,71)_ = .27, *P_ADJ_* = 0.99). This is evidence that the surface sterilization method had no effect on the hatchability of eggs compared to the control eggs. However, despite the fact that eggs were not sterilized in generation 2, the mean percent hatch of the 15 egg masses laid by adults reared from the sterilized cohorts in generation 1 was significantly lower than the hatchability of eggs from adult control cohorts (*t*_(1,71)_ = 3.73, *P_ADJ_* = .002). This was further shown by a significant interaction effect for first instars (Table 3).

For the other developmental stages during both generations, cohorts of *H. halys* from sterilized eggs or from untreated eggs laid by adults that were reared from sterilized eggs showed significantly lower survivorship than the cohorts from the control group (Figure 2, Table 3). The second generation progeny from adults of the sterilized cohorts also showed significantly lower survivorship in all developmental stages compared to the sterile cohorts in generation 1 (2^nd^ instar: *t*_(1,71)_ = 4.17, *P_ADJ_*<.001; 3^rd^ instar: *t*_(69)_ = 7.77, *P_ADJ_* = <.001; 4^th^ instar: *t*_(70)_ = 8.01, *P_ADJ_* =  <.001; 5^th^ instar: *t*_(69)_ = 6.82, *P_ADJ_* =  <.001; adults: *t*_(69)_ = 6.70, *P_ADJ_* =  <.001). Differences in survivorship between the sterilized and control cohorts depended on the generation but this interaction was not significant for all developmental stages. The treatment by generation interaction was significant for developmental stages after the second instar, except fifth instars, and the general trend showed a more pronounced lower survival of older nymphs reared from eggs of generation 1 adults of the sterilized group. The generational survival is reflected by the number of individuals that survived to the adult stage relative to the total number of eggs. Survival to adults from the control and sterilized egg masses in the first generation averaged 24.8% and 15.7%, respectively, and the difference was significant (*t*_(68)_ = 3.02, *P_ADJ_* = .018). The rate of mortality was much greater during the second generation of progeny reared from adults of the sterilized group. The average generational survival of these cohorts was only 0.3% and significantly lower than the generational survival of 20.8% for the control cohorts (*t*_(69)_ = 7.71, *P_ADJ_* =  <.0001).

### Parameters of fecundity and egg production

**Figure 3:**
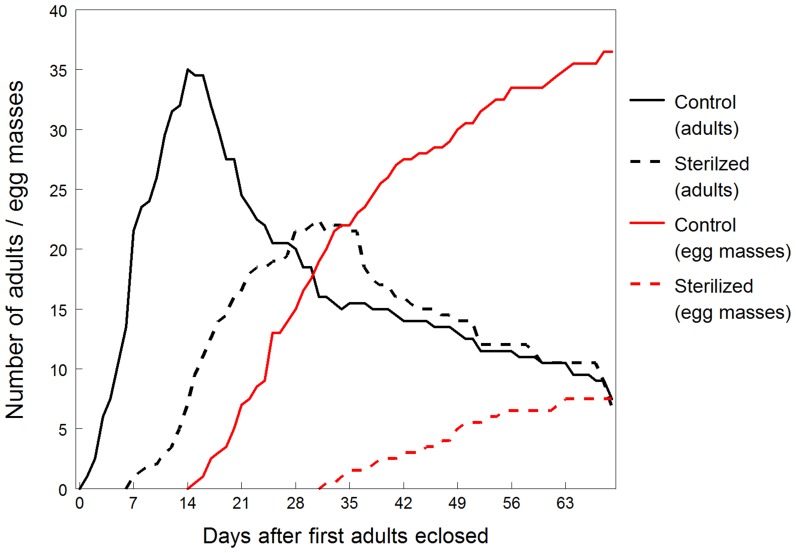
Cumulative number of generation 1 adults reared from sterilized and untreated control eggs and their egg mass production plotted over days after the first adults had eclosed.

Overall, including cage reared and PCR stored adults, Control Block A produced 38 females and 29 males, and Control Block B produced 20 females and 22 males. Sterile Block A produced 26 females and 24 males, and Sterile Block B produced 12 females and 14 males. Due to the longer developmental times of cohorts reared from surface sterilized eggs, adults were produced later and the population peaked in the rearing cages later than the control cohorts (Figure 3). In total, 46 females from the control group of generation 1 produced 73 egg masses (1.59 per female), while 29 females from the sterilized group produced 15 egg masses (0.52 per female). Females of the control group took an average of 15.5 days to begin laying eggs, while the sterilized group took 25 days to begin oviposition (Table 4). All egg masses collected from generation 1 that were not assigned to second generation cohorts were reared individually until they hatched. Data on the number of eggs per mass, days to hatch, and percent hatch grouped by treatment were compiled for these egg masses together with eggs assigned to second generation cohorts. ANOVA results showed no significant difference in hatch time between the sterilized and control treatments but females reared from the sterilized group of generation 1 produced egg masses that had 38% fewer eggs and significantly lower hatch rates compared to the egg production of control females (Table 4).

**Table 4:** Effects of surface sterilization on the fecundity of first generation adults and on the development and hatchability of second generation egg masses of *H. halys*.

*Parameter*	*Control*	*Sterile*	*df*	*F*	*P*
**Time to first egg mass (days)**	15.5±1.5	25±0	-	-	-
**Mean #egg masses/female**	1.58±0.011	0.473±0.098	-	-	-
**Mean # eggs/mass**	27.77±0.01	17.13±2.05	1, 85.3	129.52	<.0001
**Mean % hatch**	94.43±1.30	64.95±9.46	1, 85	33.44	<.0001
**Mean development time (days)**	6.42±0.08	6.50±0.11	1, 83	0.18	0.6735

### Movement behavior of the first instars

**Figure 4:**
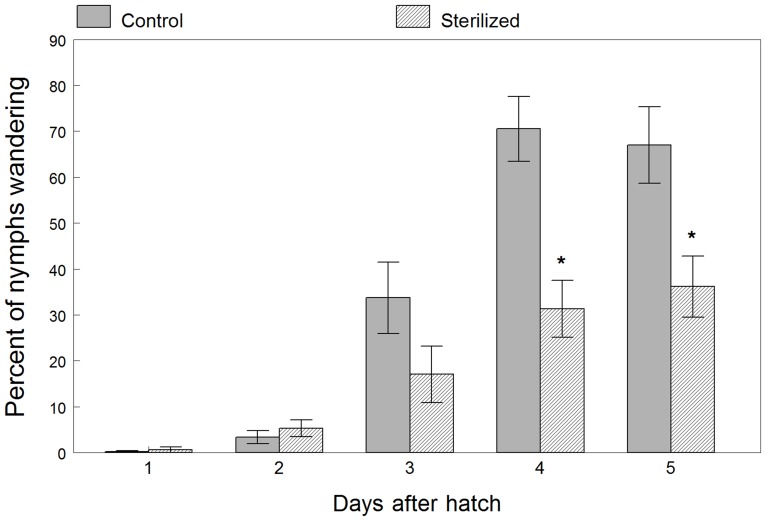
Mean percentage (± SEM) of first instars wandering away from sterilized and untreated control egg masses. An asterisk over each pair of means for each observation day indicates a significant difference (*P*<0.05).

First instar nymphs of generation 1 were observed each day for differences in their presence on and movement away from the egg mass. Similar to the methods used by Hosokawa et al. [32], the percentage of wandering nymphs was defined as the number of live nymphs not touching the leaf disc on which the egg mass was laid divided by the total number of live nymphs times 100. Differences between the control and sterilized treatments were analyzed using a two-way ANOVA, with each observation day treated as repeated measure fixed factor. Significant treatment differences in wandering behavior were observed but were not the same among the five days of observation, as evident by a significant interaction effect (Table 5). Significantly more nymphs wandered off the control egg masses during days 4 and 5 than did the nymphs on sterilized masses (Figure 4). At five days after egg hatch, 67% of the control nymphs exhibited wandering behavior in comparison to the 36% of nymphs hatching from sterilized eggs.

**Table 5:** Two-way ANOVA with percent wandering behavior as the dependent variable, egg mass treatment, day of observation after hatch, and the interaction (T x G) as fixed factors, position in the rearing chamber as a random blocking factor, and adjustment made for day as repeated measure.

*Effect of sterilization on wandering*	*Df*	*F*	*P*
**Treatment**	1, 1	129.52	<.001
**Day**	4, 168	33.44	<.001
**T x D**	1, 168	0.18	<.001

## Discussion

Results of this study provide direct evidence that *H. halys* is negatively impacted when its egg masses are surface sterilized, and this impact is significant in the first generation and manifests dramatically in the subsequent generation. In the first generation, developmental time and survivorship of treated cohorts were significantly affected during third instar development through to the adult stage. In the second generation, less than ten individuals collectively from the 15 egg masses laid by first generation adults of the sterilized group survived past the second instar, and of those only one successfully molted to adulthood. Adults from the sterilized treatment group exhibited longer pre-oviposition periods, produced fewer egg masses, had significantly smaller clutch sizes, and the hatch rate and survivorship of those eggs were significantly reduced. Taken together, this suggests that the overall fitness of *H. halys* is highly dependent on the presence of gut symbionts. These findings are consistent with the results reported by Prado and Almeida on the role of gut symbionts in the stink bug *C. hilaris* [21]. Published studies have shown that aphids rely on gut symbionts to produce certain amino acids for normal development [10], [33], and the physiological role that gut symbionts play in their host has also been reported in several heteropteran groups [13], [16], [21].

Noteworthy is the fact that the control cohorts of nymphs in the second generation experienced significantly longer developmental times and lower survival than control cohorts in the first generation. Although the rearing conditions were similar, the difference is likely due to the source of egg masses collected for the study and the different ways that they were selected for each generation. Generation 1 cohorts were selected at random from all egg masses collected on one day from several laboratory colony cages of adults which were close in age and exhibiting peak oviposition. For generation 2, egg masses were selected over several weeks from a much smaller pool of first generation adults reared from the control cohorts. We notice that egg mass production rises over a few weeks, peaks, and then decreases in our adult cages for *H. halys*. This seems to correlate to the age of the females in the cage. Egg production starts off slowly from newly reproductive females, which then increases as the adult's mature, but then tapers off at a certain point as the adults get to a certain age. The egg masses of generation 1 most likely came from middle aged females laying at their peak as opposed to the generation 2 egg masses which came from females representing different ages.

Observations were made during the first 5 days after hatch of the first generation eggs to see if treatment affected wandering behavior of the first instars. Hosokawa et al. not only draws attention to the lack of research on possible host behavior modification by a symbiont in heteropteran mutualistic relationships, but goes on to support the occurrence of this phenomenon in the species *Megacopta punctatissima* (Plataspidae) [33]. They reported a negative correlation between the supply of symbiont available and the number of wandering nymphs. In this study, the opposite behavior occurred in *H. halys*. Nymphs hatching from untreated egg masses wandered off the mass more quickly and at a higher frequency over the five days after hatch, whereas nymphs from surface sterilized eggs tended to stay on the egg mass longer. More research needs to be done to fully understand the behavior modification in nymphs when the symbiont is absent, but it is plausible that *H. halys* nymphs once adequately inoculated will move off the egg mass, while nymphs hatching from eggs without the symbiont will continue to probe longer.

Two symbionts were sequenced from the gut of *H. halys*, although the primers used in PCR failed to detect the *Wolbachia* symbiont. It is possible that the primers were sequenced from adults that carried a different strain of *Wolbachia*, or it is not present in colony individuals. More work on this symbiotic relationship is necessary. We instead focused on *P. agglomerans* due to its confirmed extracellular presence on unhatched egg masses and in the gut of colony adults. Although egg masses from the colony tested positively for *P. agglomerans*, the qualitatively weaker gel bands detected were likely due to lower quantity of the symbiont on the egg surfaces compared to levels in the gut of adults. This notion is supported by symbiont studies performed on the Mediterranean fruit fly. The fruit fly is colonized by the symbiont *P. aggolmerans*, which forms a bio-film in the gut of the fly. It migrates to the ovaries and coats the apical ends of the eggs with the same bio-film in females, albeit in a lower quantity than in the gut [34]. Pentatomids are described as smearing gut symbionts on their eggs, but it may be possible that a similar mode of action to the fruit flies is occurring. More work on this mechanism is needed. Detection of *P. agglomerans* in generation 1 adults was successful and the inoculation rate in the control group was significantly higher that that of the sterilized group. Despite the difference between treatment groups, there was evidence that the symbiont was not completely removed from the sterilized egg masses. One possible explanation may be attributed to the selection of adults sampled for DNA extraction. We unintentionally biased the selection towards the first individuals to molt to adulthood, thus these adults were likely the most fit individuals (they developed faster) and may have obtained symbionts as nymphs if any were present on the egg mass. For future studies, adults for DNA extraction should be selected across the entire population of each replicate cohort to obtain a more representative estimate of symbiont presence and to determine if time to adulthood correlates with symbiont inoculation.

Further research is needed to investigate environmental factors that affect the survival of the gut symbionts as well. It has been shown that high temperature can negatively impact symbiont survivorship and thus host life history parameters, as seen in *M. histrionica* and *C. hilaris* [25]. Preliminary studies are showing similar trends in *H. halys* when eggs are exposed to high temperatures. Abiotic factors like temperature may prove to limit the colonization capabilities of *H. halys* in certain parts of the US.

This is the first documented evidence that removal of symbionts from the surface of the egg mass results in detrimental effects on *H. halys* life history and fecundity parameters. This may have important implications in the management of this invasive pest. For instance, the high degree of reliance on the symbiont may be a weak link in its life history that could be exploited in field populations by a foliar application of a sterilizing agent targeting the eggs or through transgenic plant delivery of a specific antibiotic that interferes with the normal functioning of the symbionts in the gut of *H. halys*.

## References

[1] Leskey TC, Hamilton GC, Nielsen AL, Polk DF, Rodriguez-Saona C, et al (2012) Pest Status of the brown marmorated stink bug, *Halyomorpha halys* (Stål), in the USA. Outlooks on Pest Management 23: 218–226.

[2] Hoebeck ER, Carter ME (2003) *Halyomorpha halys* (Stål) (Heteroptera: Pentatomidae): a polyphagous plant pest from Asia newly detected in North America. Proceedings of the Entomological Society of Washington 105: 225–237.

[3] Where Is BMSB: State-by-State. *StopBMSB.org* Northeastern IPM Center Available: http://www.stopbmsb.org/where-is-bmsb/state-by-state/. Accessed 2014 Feb 9.

[4] Kuhar TP, Kamminga KL, Whalen J, Dively GP, Brust G, et al.. (2012) The pest potential of brown marmorated stink bug on vegetable crops. Plant Health Progress (Online). doi:10.1094/PHP-2012-0523-01-BR.

[5] Leskey TC, Short BD, Butler BB, Wright SE (2012) Impact of the invasive brown marmorated stink bug, *Halyomorpha halys* (Stål) in mid-Atlantic tree fruit orchards in the United States: case studies of commercial management. Psyche Article ID 535062, 14 pages. doi:10.1155/2012/535062.

[6] Hamilton GC (2009) Brown marmorated stink bug. American Entomology (Spring) 55: 19–20.

[7] Buchner P (1965) Endosymbionts of Animals with Plant Microorganisms. New York: Interscience.

[8] Moran NA, Telang A (1998) Bacteriocyte associated symbionts of insects. BioScience 48, No. 4: 295–304.

[9] Baumann P, Moran NA (1997) Non-cultivable microorganisms from symbiotic associations of insects and other hosts. Antonie van Leeuwenhoek 72: 39–48. 9296262 10.1023/a:1000239108771

[10] Douglas AE (2006) Phloem sap feeding by animals: problems and solutions. Journal of Experimental Botany 57: 747–54. 16449374 10.1093/jxb/erj067

[11] Glasgow H (1914) The gastric caeca and the caecal bacteria of the Heteroptera. Biological Bulletin 3: 101–171.

[12] Dasch GA, Weiss E, Chang KP (1984) Endosymbionts of insects. Bergey's Manual of Systematic Bacteriolog. Baltimore: Williams and Wilkins. pp. 811–833.

[13] Fukatsu T, Hosokawa T (2002) Capsule-transmitted gut symbiotic bacterium of the Japanese common plataspid stinkbug, *Megacopta punctatissima*. Applied and Environmental Microbiology 389–396. 10.1128/AEM.68.1.389-396.2002 PMC126591 11772649

[14] Hosokawa T, Kikuchi Y, Meng XY, Fukatsu T (2005) The making of symbiont capsule in the plataspid stinkbug *Megacopta punctatissima* . FEMS Microbiology Ecology 54: 471–77. 16332344 10.1016/j.femsec.2005.06.002

[15] Schorr H (1957) Zür Verhaltensbiologie und Symbiose von Brachypelta aterrima Först (Cydnidae, Heteroptera). Zeitschrift fuer Morphologie und Okologie der Tiere 45: 561–602.

[16] Abe Y, Mishiro K, Takanashi M (1995) Symbiont of brown-winged green bug, *Plautia stali* Scott. Japanese Journal of Applied Entomology and Zoology 39: 109–115.

[17] Prado SS, Rubinoff D, Almeida RPP (2006) Vertical transmission of a pentatomid caeca-associated symbiont. Annals of the Entomological Society of America 99: 577–585.

[18] Kikuchi Y, Hosokawa T, Nikoh N, Meng XY, Kamagata Y, et al (2009) Host-symbiont co-speciation and reductive genome evolution in gut symbiotic bacteria of acanthosomatid stinkbugs. BMC Biology 7: 2. 19146674 10.1186/1741-7007-7-2 PMC2637841

[19] Kaiwa N, Hosokawa T, Kikuchi Y, Nikoh N, Meng XY, et al (2010) Primary gut symbiont and secondary, *Sodalis*-allied symbiont in the scutellerid stinkbug *Cantao ocellatus* . Applied Environmental Microbiology 76: 3486–3494. 20400564 10.1128/AEM.00421-10 PMC2876435

[20] Kikuchi Y, Hosokawa T, Fukatsu T (2007) Insect-microbe mutualism without vertical transmission: a stinkbug acquires a beneficial gut symbiont from the environment every generation. Applied and Environmental Microbiology 4308–4316. 10.1128/AEM.00067-07 PMC1932760 17483286

[21] Prado SS, Almeida RPP (2009) Role of symbiotic gut bacteria in the development of *Acrosternum hilare* and *Murgantia histrionica* . Entomological Experimental Applications 132: 21–29.

[22] Kikuchi Y, Hosokawa T, Nikoh N, Fukatsu T (2012) Gut symbiotic bacteria in the cabbage bugs *Eurydema rugosa* and *Eurydema dominulus* (Heteroptera: Pentatomidae). Applied Entomology and Zoology 47: 1–8.

[23] Kashima T, Nakamura T, Tojo S (2006) Uric acid recycling in the shield bug, *Parastrachia japonensis* (Hemiptera: Parastrachiidae) during diapause. Journal of Insect Physiology 52: 816–825. 16797581 10.1016/j.jinsphys.2006.05.003

[24] Hosokawa T, Hironaka M, Mukai H, Inadomi K, Suzuki N, et al (2012) Mothers never miss the moment: a fine-tuned mechanism for vertical symbiont transmission in a subsocial insect. Animal Behavior 83: 293–300.

[25] Prado SS, Hung KY, Daugherty MP, Almeida RPP (2010) Indirect effects of temperature on stink bug fitness, via maintenance of gut-associated symbionts. Applied and Environmental Microbiology 1261–1266. 10.1128/AEM.02034-09 PMC2820946 20023083

[26] Prado SS, Almeida RPP (2009) Phylogenetic placement of pentatomid stink bug gut symbionts. Current Microbiology 58: 64–69. 18810535 10.1007/s00284-008-9267-9

[27] Hosokawa T, Kikuchi Y, Nikoh N, Fukatsu T (2012) Polyphyly of gut symbionts in stinkbugs of the family Cydnidae. Applied Environmental Microbiology 78: 4758–4761. 22544238 10.1128/AEM.00867-12 PMC3370497

[28] Kaiwa N, Hosokawa T, Kikuchi Y, Nikoh N, Meng XY, et al (2011) Bacterial symbionts of the Giant Jewel Stinkbug *Eucorysses grandis* (Hemiptera: Scutelleridae). Zoological Science 28: 169–174. 21385056 10.2108/zsj.28.169

[29] Prado SS, Zucchi TD (2012) Host-symbiont interactions for potentially managing Heteropteran pests. Psyche 2012: article ID 269473. 9 p.

[30] Nielsen AL, Hamilton GC, Matadha D (2008) Developmental rate estimation and life table analysis for *Halyomorpha halys* (Hemiptera: Pentatomidae). Environmental Entomology 27: 348–355. 10.1603/0046-225x(2008)37[348:drealt]2.0.co;2 18419906

[31] Lockwood JA, Story RN (1998) Photic, thermic, and sibling influences on the hatching rhythm of the southern green stink bug, *Nezara viridula* (L.). Environmental Entomology 14: 562–567.

[32] Hosokawa T, Kikuchi Y, Shimada M, Fukatsu T (2008) Symbiont acquisition alters behavior of stinkbug nymphs. Biological Letters 4: 45–48. 10.1098/rsbl.2007.0510 PMC2412937 18055411

[33] Douglas AE (1998) Nutritional interactions in insect-microbial symbioses: Aphids and their symbiotic bacteria *Buchnera* . Annual Review of Entomology 43: 17–37. 10.1146/annurev.ento.43.1.17 15012383

[34] Lauzon CR, Mccombs SD, Potter SE, Peabody NC (2009) Establishment and vertical passage of *Enterobacter (Pantoea) Agglomerans* and *Klebsiella pneumoniae* through all life stages of the Mediterranean fruit fly (Diptera: Tephritidae). Annals of the Entomological Society of America 102(1): 85–95.

